# Extraction, Isolation and Biological Activity of Two Glycolipids from *Bangia fusco-purpurea*

**DOI:** 10.3390/md22040144

**Published:** 2024-03-24

**Authors:** Yingying Sun, Yang Mu, Tianhuan Li, Siyu Wang, Yuxiang Li, Jie Liu, Piaopiao Xing

**Affiliations:** 1 Jiangsu Key Laboratory of Marine Bioresources and Eco-Environment, Jiangsu Ocean University, Lianyungang 222005, China2022220838@jou.edu.cn (T.L.); 2021122091@jou.edu.cn (S.W.); 2021122119@jou.edu.cn (J.L.);; 2 Jiangsu Institute of Marine Resources Development, Lianyungang 222005, China; 3 Co-Innovation Center of Jiangsu Marine Bio-Industry Technology, Lianyungang 222005, China; 4 Jiangsu Key Laboratory of Marine Biotechnology, Jiangsu Ocean University, Lianyungang 222005, China

**Keywords:** *Bangia fusco-purpurea*, extraction, glycolipids, isolation, marine macroalgae, moisture-absorption activity

## Abstract

In order to explore the extraction and activity of macroalge glycolipids, six macroalgae (*Bangia fusco-purpurea*, *Gelidium amansii*, *Gloiopeltis furcata*, *Gracilariopsis lemaneiformis*, *Gracilaria* sp. and *Pyropia yezoensis*) glycolipids were extracted with five different solvents firstly. Considering the yield and glycolipids concentration of extracts, *Bangia fusco-purpurea*, *Gracilaria* sp. and *Pyropia yezoensis* were selected from six species of marine macroalgae as the raw materials for the extraction of glycolipids. The effects of the volume score of methanol, solid–liquid ratio, extraction temperature, extraction time and ultrasonic power on the yield and glycolipids concentration of extracts of the above three macroalgae were analyzed through a series of single-factor experiments. By analyzing the antioxidant activity in vitro, moisture absorption and moisturizing activity, the extraction process of *Bangia fusco-purpurea* glycolipids was further optimized by response surface method to obtain suitable conditions for glycolipid extraction (solid-liquid ratio of 1:27 g/mL, extraction temperature of 48 °C, extraction time of 98 min and ultrasonic power of 450 W). *Bangia fusco-purpurea* extracts exhibited a certain scavenging effect on DPPH free radicals, as well as good moisture-absorption and moisture retaining activities. Two glycolipids were isolated from *Bangia fusco-purpurea* by liquid–liquid extraction, silica gel column chromatography and thin-layer chromatography, and they showed good scavenging activities against DPPH free radicals and total antioxidant capacity. Their scavenging activities against DPPH free radicals were about 60% at 1600 µg/mL, and total antioxidant capacity was better than that of Trolox. Among them, the moisturizing activity of a glycolipid was close to that of sorbierite and sodium alginate. These two glycolipids exhibited big application potential as food humectants and antioxidants.

## 1. Introduction

Glycolipids are a class of compounds formed by one or more monosaccharide residues linked to lipid moieties, monoacyl or diallylglycerols by glycosidic bonds [1]. They widely exist in various organisms. However, compared to other natural compounds, such as terpenoids, glycolipids are neglected for a long time [2]. Recently, with the prominence of ecological or pharmaceutical interest activities, the interest in glycolipids of marine origin such as marine macro-micro algae and microorganism has grown rapidly.

The existence of hydrophilic sugar group and lipophilic acyl group determines that glycolipids are amphoteric compounds. And this special amphiphilic gives them special physiological activities. Glycolipids from *Sargassum vulgare* had good antifouling activity [3]. A glycolipid isolated from *Lobophora variegata* showed significant allelopathic activity against the coral *Montastraea cavernosa* and the sponge *Agelas clathrodes* [4]. Glycolipids from macroalgae presented antibacterial [5,6], antiviral [7,8], anti-inflammatory [9], antioxidant [10], anticancer [11], antifouling activities [3], antialgal activity [12] and other activities [13,14]. Among these studies mentioned above, the moisture-absorption and moisture-retention activities of glycolipids from marine macroalgae have received less attention.

It has been reported that the sugar of glycolipids is mainly galactose [8,15], followed by sulfo-rhamnose [5,8,15] and glucose exists only in very few glycolipids from some marine macroalgae [16]. For example, the sugars of glycolipids from *Ascophyllum nodosum* [17], *Grateloupia turuturu* [18], *Sargassum thunbergii* [6], *Caulerpa racemosa* [8], *Chondria armata* [5], *Palmaria palmata* [15] and *Sargassum fulvellum* [16] were galactose, sulfo-rhamnose or glucose. The sugars of the two glycolipids isolated from *Pelvetia siliquosa* and *Porphyra haitanensis* in our previous study were also galactose [12]. Up to now, arabinose has not bn in the glycolipids from seaweeds.

Although the isolation and purification technology are becoming more and more mature, the isolation and purification of glycolipids is still difficult due to the similarity of their structures. Currently, a completely single natural standard glycolipid is not available from the chemical market, and chemical companies are only able to offer the mixed glycolipid standards for TLC purity, which are expensive. In order to break this status of glycolipid supply and promote the application of glycolipids in industries such as food, medicine and cosmetics, the glycolipids of more macroalgae need to be extracted, isolated and purified. In the present study, *Bangia fuscopurpurea* (Dillwyn, Lyngbye, 1819), *Gelidium amansii* (J.V.Lamouroux, 1813), *Gloiopeltis furcate* (Postels and Ruprecht, J. Agardh, 1851), *Gracilariopsis lemaneiformis* (Bory, E.Y.Dawson, Acleto and Foldvik, 1964), *Gracilaria* sp., *Palmaria palmata* (Lannaeus, Weber and Mohr, 1805) and *Pyropia yezoensis* (Ueda, M. S. Hwang and H. G. Choi, 2011) were selected as raw materials for extracting glycolipids to obtain new types of glycolipids from these marine macroalgae, and find their activities. As far as we know, there have been no reports on the extraction of glycolipids of the six macroalgae. Also, we studied the moisture-absorption and moisture-retention activities of glycolipids from marine macroalgae to see if they could be developed as natural humectants.

## 2. Results

### 2.1. Effects of Different Solvents and Extraction Times on the Yields of Six Macroalgae Extracts

**Figure 1:**
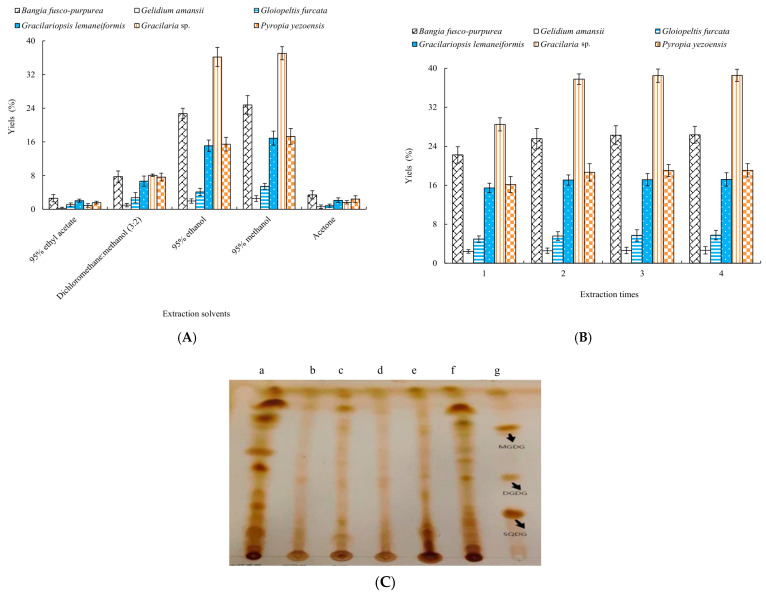
Effects of different extraction solvents (**A**) and extraction times (**B**) on the yields of six macroalgae extracts and TLC of 95% methanol extracts (a. *Bangia fusco-purpurea*; b. *Gelidium amansii*; c. *Gloiopeltis furcata*; d. *Gracilariopsis lemaneiformis*; e. *Gracilaria* sp.; f. *Pyropia yezoensis*; g. glycolipid standards) of six macroalgae (**C**). The data in the figure are expressed as the mean ± SD.

Different solvents had significant (*p* < 0.05) effects on the yields of six macroalgae extracts (Figure 1A). It could be clearly seen from Figure 1 that the higher yields could be obtained using 95% ethanol and 95% methanol as the extraction solvents; notably, the highest yields of six macroalgae extracts were obtained with 95% methanol as the extraction solvent. The yields of *Bangia fusco-purpurea*, *Gelidium amansii*, *Gloiopeltis furcata*, *Gracilariopsis lemaneiformis*, *Gracilaria* sp. and *Pyropia yezoensis* extracts were 20.8%, 2.37%, 5.21%, 11.8%, 30.2% and 13.8%, respectively.

In Figure 1B, as the extraction time increased, the yields of six macroalgae extracts increased. When the extraction times exceeded two times, the increase in the yields of six macroalgae extracts was not significant (*p* > 0.05). Moreover, 2~3 extraction times were more suitable for the extraction of six macroalgae.

Thin-layer chromatography of 95% methanol extracts of six macroalgae was determined by a silica gel G plate (Figure 1). The macroalgae extracts all showed positive reactions for glycolipids. Notably, a series of very obvious spots appeared in the three extracts of *Bangia fusco-purpurea*, *Gracilaria* sp. and *Pyropia yezoensis*. Considering the yield and TLC results of extracts, the extraction processes of glycolipids of these three macroalgae were analyzed by single-factor experiments in the following experiments.

### 2.2. Single-Factor Experiments

**Figure 2:**
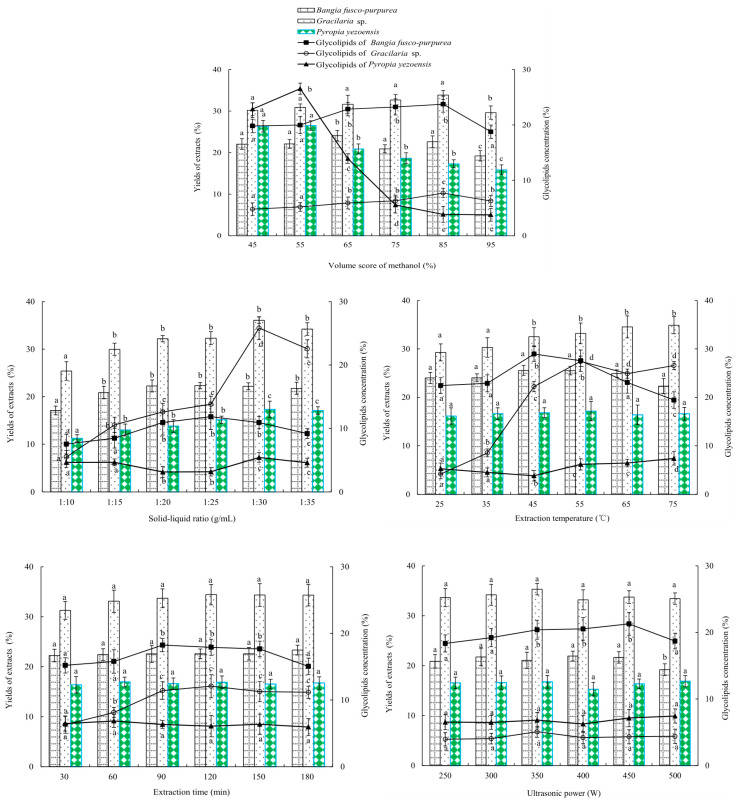
Effects of different factors and factor levels on the yield of extract and the glycolipids contents in extracts from 6 species of marine macroalgae in single-factor experiments. Different letters (a, b, c, d and e) indicate significant differences. The data in the figure are expressed as the mean ± SD.

In Figure 2, effects of the volume score of methanol on the yields and glycolipids concentration of *Bangia fusco-purpurea*, *Gracilaria* sp. and *Pyropia yezoensis* extracts were similar, that is, as the volume score of methanol increased, the yields and glycolipids concentration of the three macroalgae extracts first increased and then significantly decreased (*p* < 0.05). At the volume scores of methanol of 85%, 85% and 55%, the yields and glycolipids concentration of *Bangia fusco-purpurea*, *Gracilaria* sp. and *Pyropia yezoensis* extracts reached its maximum value, which were 22.623% and 23.748%, 33.860% and 7.698%, 26.520% and 26.549%, respectively.

The solid–liquid ratio significantly (*p* < 0.05) influenced the yields and glycolipids concentration of th *Bangia fusco-purpurea*, *Gracilaria* sp. and *Pyropia yezoensis* extracts (Figure 2). With the increase of the solid–liquid ratio, the yield and concentration first increased and then tended to be stable. When the solid–liquid ratio was 1:25 g/mL, 1:30 g/mL and 1:30 g/mL, the yields and glycolipids concentration of the *Bangia fusco-purpurea*, *Gracilaria* sp. and *Pyropia yezoensis* extracts reached maximums, which were 22.316% and 11.838%, 36.080% and 25.821%, 17.310% and 5.438%.

Extraction temperature showed a significant (*p* < 0.05) effect on the glycolipids concentration of *Bangia fusco-purpurea* and *Gracilaria* sp. extracts, but had no significant (*p* > 0.05) effect on the yields of the two macroalgae extracts (Figure 2). At 45 °C and 55 °C, the glycolipids concentrations of the *Bangia fusco-purpurea* and *Gracilaria* sp. extracts reached their maximums 28.959% and 27.439%. The yield and glycolipids concentration of the *Pyropia yezoensis* extracts showed different trends; the former increased with the increase of extraction temperature, and the latter significantly (*p* < 0.05) decreased first and then significantly (*p* < 0.05) increased with the increase of extraction temperature.

From Figure 2, extraction time and ultrasonic power did not significantly (*p* > 0.05) affect the yields of three macroalgae extracts, but only had a significant (*p* < 0.05) effect on the glycolipids concentration of *Bangia fusco-purpurea* (or *Gracilaria* sp.) extracts. The extraction time and ultrasonic power of 90 min and 450 W, respectively, were suitable for the extraction of glycolipids of the three macroalgae.

In the extraction process of glycolipids of *Bangia fusco-purpurea*, *Gracilaria* sp. and *Pyropia yezoensis*, the solid-to-liquid ratio, extraction temperature, extraction time and ultrasonic power were important factors (Figure 2). Therefore, in the optimization experiments by response surface methodology, the effects of these four factors were further analyzed on the yield of extracts and glycolipids concentration of marine macroalgae. To reduce the workload, it was necessary to select one macroalgae from the above three macroalgae and study only its glycolipids extraction by the response surface experiment. The antioxidant, moisture-absorption and moisture-retaining activities of *Bangia fusco-purpurea*, *Gracilaria* sp. and *Pyropia yezoensis* extracts were studied in the following experiments in order to obtain target macroalgae.

### 2.3. Properties of Antioxidant, Moisture-Absorption and Moisture-Retaining

#### 2.3.1. Antioxidant Activity

**Figure 3:**
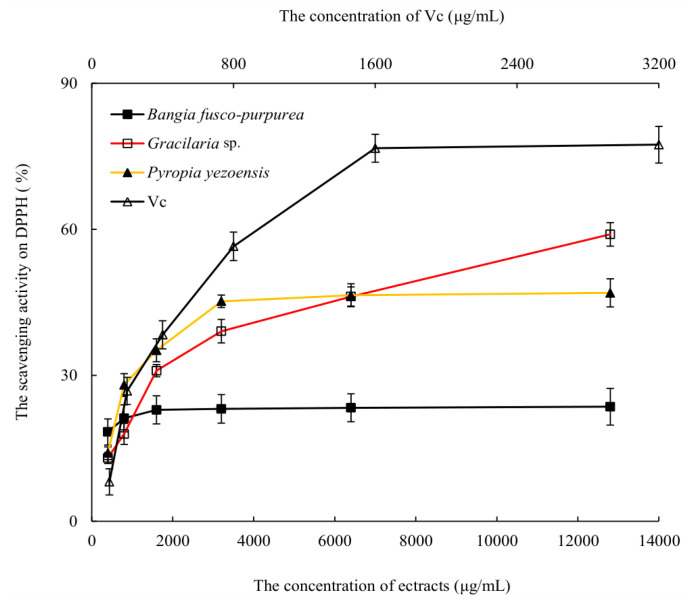
The scavenging activities of three macroalgae extracts activity on DPPH free radicals. The data in the figure are expressed as the mean ± SD.

The scavenging activities of three macroalgae extracts against DPPH free radicals were analyzed. With the increase of the extracts’ concentration, the scavenging abilities of DPPH free radicals were gradually enhanced, and the scavenging activities no longer increased significantly (*p* > 0.05) when the extracts’ concentration was greater than 3200 μg/mL (Figure 3). Among them, the scavenging abilities of *Gracilaria* sp. and *Pyropia yezoensis* extracts were higher than that of *Bangia fusco-purpurea* extracts. The scavenging abilities of these macroalgae extracts on DPPH free radicals were significantly (*p* < 0.05) better than that of the polysaccharide extracts of *Gelidium amansii* and *Ecklonia kurome*, and the maximum scavenging ability of the latter on DPPH free radicals reached about 25% at 8 mg/mL [19]. Vitamin C (Vc) had a strong scavenging ability to DPPH free radicals, and the scavenging ability of Vc was always stronger than that of these three macroalgae extracts at the same concentration, and the scavenging activity of Vc against DPPH free radicals had reached more than 76% when the concentration was 1600 μg/mL.

Although the scavenging activities of three macroalgae extracts on DPPH free radicals were significantly lower than that of Vc, it may be due to the fact that these were currently crude extracts, and the scavenging activities against DPPH free radicals may be significantly enhanced when the purity of these extracts was improved.

#### 2.3.2. Moisture-Absorption and Moisture-Retaining Activity

**Figure 4:**
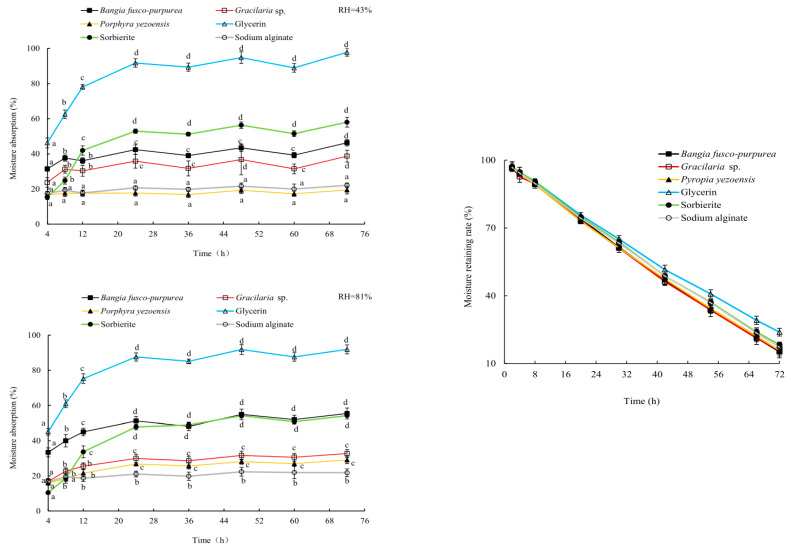
The properties of moisture absorption (a, b, c, and d indicate significant differences) and moisture retaining of three macroalgae extracts. The data in the figure are expressed as the mean ± SD.

Glycerin, sorbierite and sodium alginate are commonly used moisture-absorption and moisture-retaining agents [20,21], and they were used here as controls in this study. Three macroalgae extracts showed good moisture absorption activities, which were significantly (*p* < 0.05) higher than or very close (*p* > 0.05) to that of sodium alginate at relative humidity (RH) of 43% and 81%. Among them, *Bangia fusco-purpurea* extracts exhibited the best moisture-absorption activity, which even exceeded the moisture-absorption activity of sorbierite at RH of 81% (Figure 4). At 72 h, the moisture-absorption activity of *Bangia fusco-purpurea* extracts exceeded 45%. The moisture-adsorption activity of *Laminaria japonica* extracts [22] was significantly (*p* < 0.05) lower than that of *Bangia fusco-purpurea* extracts in this work.

In the first 42 h, the moisturizing rates of *Bangia fusco-purpurea*, *Gracilaria* sp. and *Pyropia yezoensis* extracts were very close (*p* > 0.05) to that of glycerin, sorbierite and sodium alginate. After 54 h, their moisturizing rate was markedly (*p* < 0.05) lower than that of glycerin, while was still very close (*p* > 0.05) to that of sodium alginate (Figure 4). At 72 h, the moisturizing rates of *Bangia fusco-purpurea*, *Gracilaria* sp. and *Pyropia yezoensis* extracts were maintained at 15%, and their moisturizing activities were significantly (*p* < 0.05) higher than that of the polysaccharide extracts of *Gelidium amansii* and *Ecklonia kurome* for 24 h [19]. There was no significant (*p* > 0.05) difference in the moisturizing activity of these three extracts during the whole experimental period.

**Figure 5:**
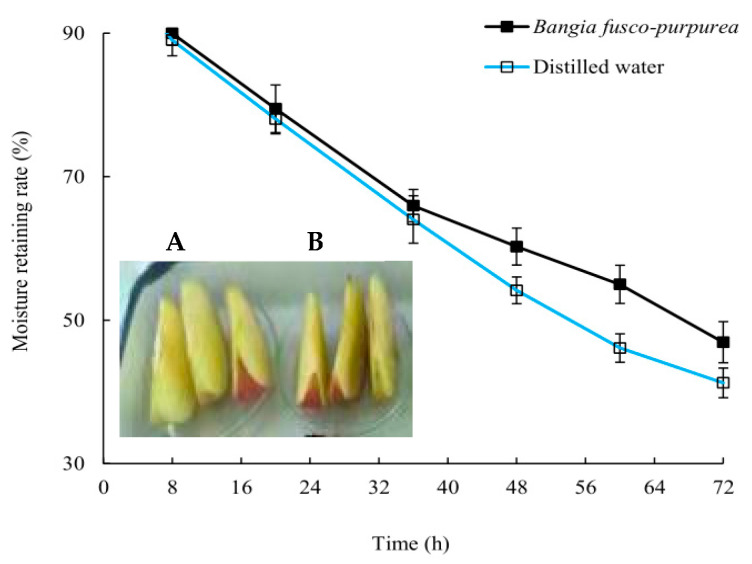
The properties of moisture retaining of *Bangia fusco-purpurea* extracts (**A**) on the apple pieces. (**B**) represents the control group. The data in the figure are expressed as the mean ± SD.

After 48 h, the moisturizing rate of the apple pieces soaked in *Bangia fusco-purpurea* extracts and distilled water began to show a significant (*p* > 0.05) difference, and this difference continued until the end of the experiment (Figure 5). Finally, the moisturizing rate of apple pieces decreased from 94.97% to 41.25% in the control group and to 46.92% in the experimental group. In addition, it was also found that the browning of apple pieces soaked in *Bangia fusco-purpurea* extracts was significantly (*p* < 0.05) weaker than that of the control group after 48 h, which should be related to the antioxidant activity of *Bangia fusco-purpurea* extracts.

#### 2.3.3. Antibacterial Activity

**Figure 6:**
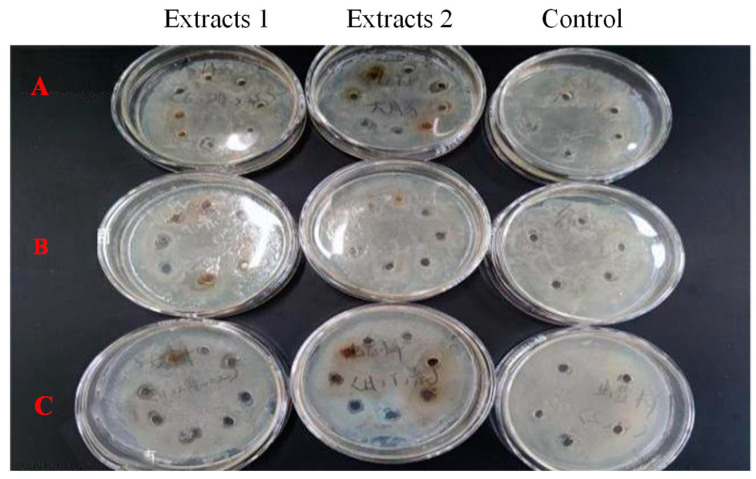
Effects of *Bangia fusco-purpurea* extracts on the *Escherichia coli* (**A**), *Shewanella putrefaciens* (**B**) and *Bacillus cereus* (**C**). Extracts 1 and 2 indicated the concentration of 25 mg/mL and 6.4 mg/mL, respectively. The experimental group with the addition of macroalgae extracts was set up with 8 parallel samples, and the control group was set up with 5 parallel samples.

At the concentration of 25 mg/mL and 6.4 mg/mL, *Bangia fusco-purpurea* extracts did not show antibacterial activity against *Escherichia coli*, *Shewanella putrefaciens* and *Bacillus cereus* (Figure 6), which are common harmful bacteria in food.

To sum up, the glycolipids concentration, the moisture-absorption and moisture retaining activities of *Bangia fusco-purpurea* extracts were superior to the other two macroalgae (Table 1), and the yield of the extracts and scavenging ability to DPPH free radicals in vitro were not very low (Figure 3), so the follow-up response surface optimization extraction process of *Bangia fusco-purpurea* extracts was carried out.

### 2.4. Response Surface Experiments

Response surface methodology (RSM) is a commonly used optimization method for analysis. The optimal conditions are predicted by response surface analysis [23], for example, to find the optimum conditions to valorize chestnut shell bioactive compounds [24], or to optimize the extraction process antioxidant extracts from brown algae *Ascophyllum nodosum* [25]. On the basis of the single-factor tests, the yield of extracts was used as the response value, and the four factors (solid–liquid ratio, extraction temperature, extraction time and ultrasonic power) and three levels of response surface analysis experiments were carried out, and the results are shown in Table 2.

**Figure 7:**
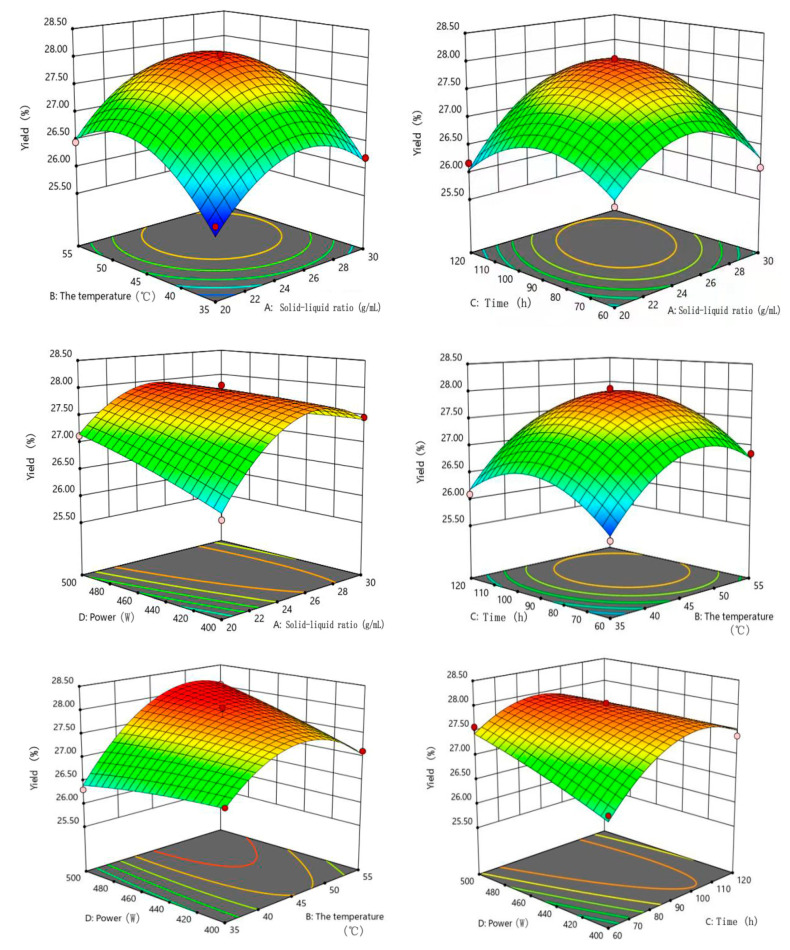
Effects of the solid–liquid ratio, temperature, time and ultrasonic power on the yield of extracts of *Bangia fusco-purpurea* in response surface analysis experiments. The red dots in the figure represent extreme values.

In order to better study the influence of the interactions between the factors and the yield of extracts, the three-dimensional surface diagram and contour plot of the response surface were drawn (Figure 7). From Figure 7, the linear parameters A (solid-to-liquid ratio), B (extraction temperature), C (extraction time), D (ultrasonic power), quadratic parameters (A^2^, B^2^ and C^2^), the interaction between solid-to-liquid ratio and extraction time (AC), the interaction between solid-to-liquid ratio and ultrasonic power (AD),the interaction between extraction temperature and ultrasonic power (BD) and the interaction between extraction time and ultrasonic power (CD) significantly (*p* < 0.01 or *p* < 0.05) affected the yield of extracts from *Bangia fusco-purpurea*. Further, Design-Expert 11.0 statistical software was used to fit the data in Table 2 by multiple regression equation, and the best fitting quadratic regression equation for the yield (*Y*_yield_) was determined as follows:

*Y*_yield_ = 27.9 + 0.3133A + 0.5000B + 0.2075C + 0.1775D + 0.0400AB + 0.3175AC − 0.2675AD + 0.0475BC + 0.3425BD − 0.2925CD − 0.7737A^2^ − 0.7212B^2^ − 0.6150C^2^ − 0.575D^2^ (1)

The analysis of variance was performed on regression Equation (1) for the yield of extracts. According to Table 3, *p* value of the model was less than 0.0001, indicating that the difference of regression Equation (1) was extremely significant, and *p* = 0.8516 of lack of fit was greater than 0.05, indicating that the agreement of the equation was good and the model was established. The correlation coefficient (*R*^2^ = 0.9910) and the correction factor (*R*_Adj_^2^ = 0.9820) indicated that the yield in high agreement with the model, which could well reflect the relationships between the yield and these four factors (solid-to-liquid ratio, extraction temperature, extraction time and ultrasonic power). The coefficient of variability CV was 0.3470%, indicating that the experiment was stable. Meanwhile, it could be concluded that the influence of each factor on the yield of extracts was extraction temperature (B) > solid–liquid ratio (A) > extraction time (C) > ultrasonic power (D) according to *F*-value in Table 2.

According to the regression model analysis (regression Equation (1)), the optimal extraction conditions for the yield were obtained using Design-Expert 11.0 statistical software. The optimum yield of 28.020% ± 0.0568% was predicted at the solid-to-liquid ratio of 1:27.46 g/mL, extraction temperature of 47.55 °C, extraction time of 98.18 min and ultrasonic power of 452.39 W. Considering the actual situation, the solid–liquid ratio, extraction temperature, extraction time and ultrasonic power were set to 1:27 g/mL, 48 °C, 98 min and 450 W, respectively. The average yield of the extracts was 28.090% ± 1.921%, and this value was close to the predicted value (28.020% ± 0.0568%), indicating that the model was stable and reliable.

Compared to the results of single factor experiments, the yield of extracts significantly improved. For example, the maximum yield of extracts in single-factor experiments was 25.57% with the solid-to-liquid ratio of 1:25 g/mL, extraction temperature of 45 °C, extraction time of 120 min and ultrasonic power of 500 W (Figure 1). The results showed that the optimization experiments of extraction process of *Bangia fusco-purpurea* extracts were necessary by response surface methodology (RSM).

### 2.5. Isolation and Purification

In order to isolate glycolipids from *Bangia fusco-purpurea* extracts, the extracts were first portioned by liquid–liquid extraction with different solvents. In this portion process, when the extracts were dissolved in a buffer solution with pH 2, the yields of fractional portions (HP, DP, EP and BP) from the extracts were higher than or close to those obtained when the extracts were dissolved in distilled water (Table 4), especially the yields of HP and DP. This should be related to the solubility of these portions in water or buffer. The buffer solution was pH 2.

So, these *Bangia fusco-purpurea* extracts were dissolved using buffer solution with pH 2 in following experiments. The total yields of these portions isolated from *Bangia fusco-purpurea* extracts were 15.487% ± 4.415%.

**Figure 8:**
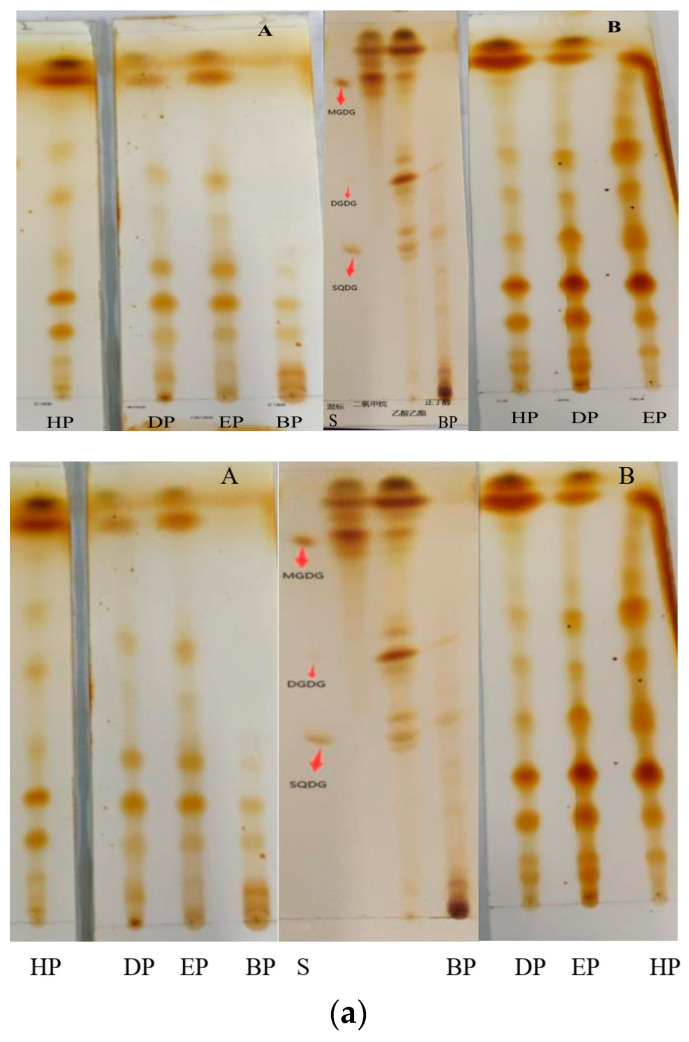

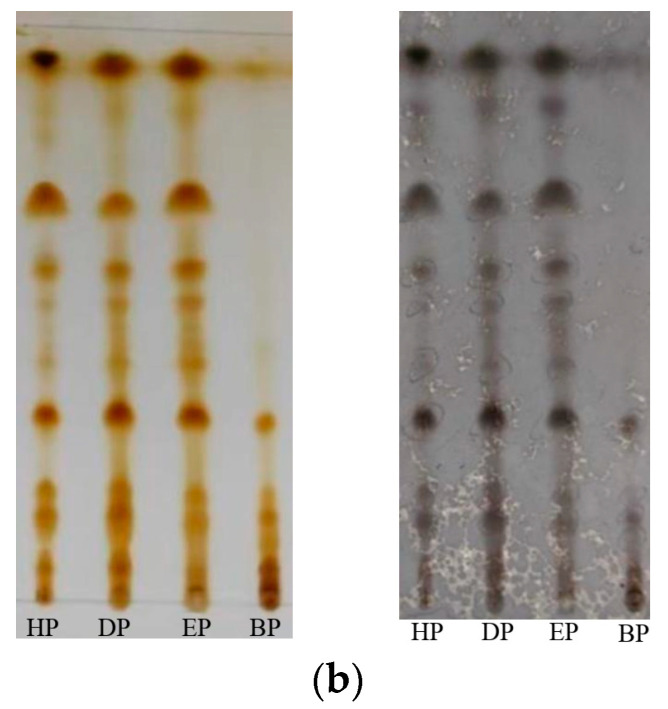
(**a**) Thin-layer chromatography of several portions extracted from *Bangia fusco-purpurea* extracts by liquid–liquid extraction. The extracts were dissolved in distilled water (**A**) and buffer solution (**B**), respectively; (**b**) Thin-layer chromatography of the four portions with different chromogenic agents. These portions were extracted from *Bangia fusco-purpurea* extracts, which were dissolved in buffer solution. HP, DP, EP, BP and S represent the hexane portion, dichloromethane portion, ethyl acetate portion, n-butanol portion and mix standard glycolipids, respectively.

Further, the behaviors of the two kinds of portions obtained by the same solvent on silica gel plates were similar (Figure 8a). And except for to BP, which did not achieve a particularly good, developed effect, the others portions were well separated, and the spots and the spacing between spots were clear (Figure 8b).

**Figure 9:**
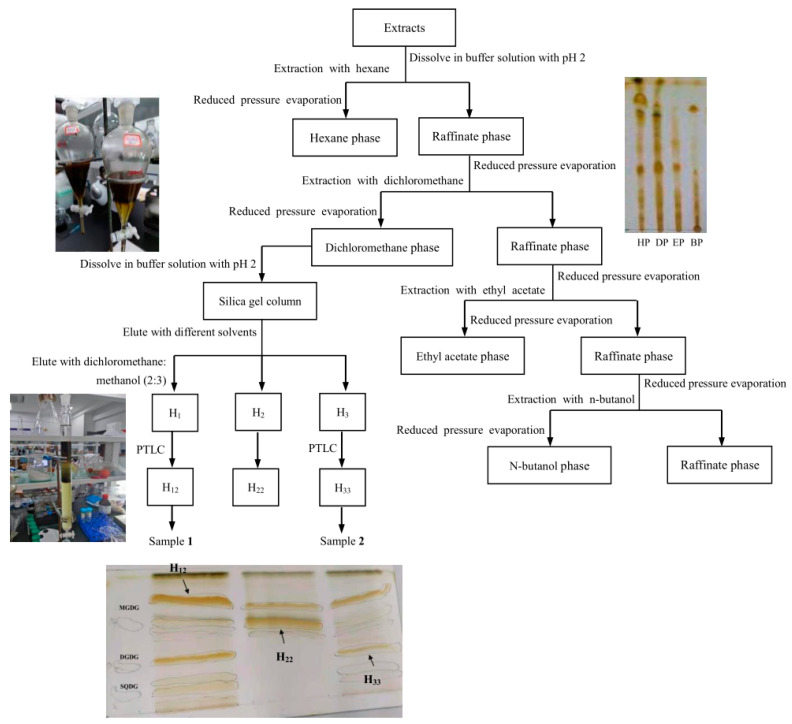
The scheme of extraction, isolation and purification of H_12_ and H_33_.

According to the yields of the portions in Table 3 and their developments in thin-layer chromatography, DP was selected for subsequent isolation using silica gel column chromatography (2.0 × 40 cm, 100–200 mesh), and three fractions (H_1_, H_2_ and H_3_) were obtained when dichloromethane/methanol (2:3, *v*:*v*) was used as eluent, specifically tube 1~tube 18 (0.853 g) for H_1_, tube 19~tube 36 (1.146 g) for H_2_ and tube 37~tube 45 (2.647 g) for H3 (Supplementary Material Figure S1). TLC determination showed that there three fractions containing glycolipids. Furthermore, H_1_, H_2_ and H_3_ were purified by preparation thin-layer chromatography. In Supplementary Material Figure S2, it could be clearly seen that the strips of H_12_, H_22_ and H_33_ were not in the same positions as that of the three standards MGDG, DGDG and SQDG in PTLC. Finally, H_12_ (20.1 mg), H_22_ (25.6 mg) and H_33_ (33.6 mg) were prepared. The above isolation and purification process is shown in Figure 9.

### 2.6. Activity Analysis

**Figure 10:**
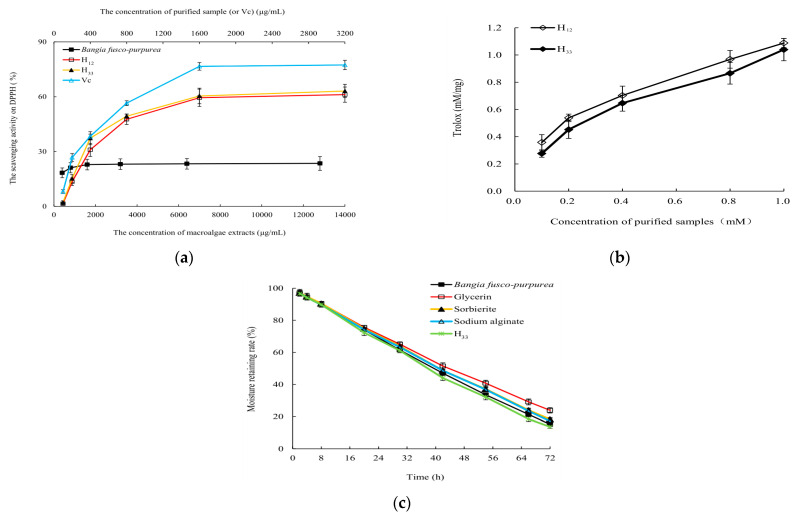
The scavenging activity to DPPH free radicals (**a**), total antioxidant capacity (**b**) and moisture-retaining activity (**c**) of H_12_ and H_33_ obtained from *Bangia fusco-purpurea* extracts. The data in the figure are expressed as the mean ± SD.

Due to the small amount of H_12_ and H_33_, they were analyzed for the scavenging activities on DPPH free radicals and TEAE (Figure 10a), and only H_33_ was analyzed for moisturizing activity (Figure 10b). Results showed that H_12_ and H_33_ showed good antioxidant activities in vitro. At 1600 µg/mL, they had scavenging rate of about 60% for DPPH free radical. There was no significant (*p* > 0.05) difference in the ability of H_12_ and H_33_ to scavenge DPPH free radicals. Compared with *Bangia fusco-purpurea* extracts, the ability of these two purified samples to scavenge DPPH free radicals in vitro was significantly (*p* < 0.01) enhanced. The half-maximal inhibitory concentrations (IC_50_) values of H_12_ and H_33_ for DPPH free radicals were 979.1 µg/mL and 877.8 µg/mL, respectively. From Figure 10b, the total antioxidant capacity of H_12_ and H_33_ was higher than that of Trolox at the tested concentration. TEAC of 0.1 mM of H_12_ (or H_33_) was 0.358 mM Trolox (or 0.2764 mM Trolox).

The moisturizing rate of H_33_ was very close (*p* < 0.05) to that of glycerin, sorbierite and sodium alginate in the first 30 h. Over time, although the moisturizing effect of H_33_ began to be weaker than that of glycerol, the moisturizing effect was very close (*p* < 0.05) to that of sorbierite and sodium alginate within the first 60 h. During the whole experiment, there was no significant difference (*p* > 0.05) between the moisturizing effects of H_33_ and *Bangia fusco-purpurea* extracts (Figure 10c).

## 3. Discussion

Glycolipids are compounds in which sugars are linked to glycerides by glycosidic bonds, which have diverse structures with chiral principles. Due to the reversibility of the esterification reaction and the large number of hydroxyl groups on the sugar molecule, the chemical synthesis conditions of glycolipids were poor, the selectivity was low and there were many by-products [26,27]. Compared with chemical synthesis, enzyme-catalyzed synthesis of glycolipids had the advantages of mild reaction conditions, high efficiency, strong selectivity and few by-products, but the types of sugar groups used in the synthesis were mostly glucose [28], galactose [29], sucrose [30], fructose and maltose [31], etc., and few glycolipid types were obtained. Up to now, the isolation and purification have been the primary and convenient ways to obtain glycolipids, especially as the isolation and identification technology have become more and more mature.

Glycolipids are widely present in living organisms. Among the many sources of glycolipids, marine macroalgae has become one of the ideal sources of natural glycolipids due to its diverse species and stable production. For example, monogalactosyl diacylcerol (MGDG) [12], monogalactosyl monoacylglycerol (MGMG) [32], digalactosyl diacylglycerol (DGDG) [3], digalactosyl monoacylglycerol (DGMG) [12], sulfoquinovosyl diacylglycerol (SQDG) [3], sulfoquinovosyl monoacylglycerol (SQMG) [33], diacylglycerols POGG (1-*O*-palmitoyl-2-*O*-oleoyl-3-*O*-(α-D-glucopyranosyl) glycerol) and MOGG (1-*O*-myristoyl-2-*O*-oleoyl-3-*O*-(α-D-glucopyranosyl) glycerol) [16] and other types of monosaccharide monoacylglycerols, such as 1-*O*-myristoyl-3-*O*-(6′-sulfo-α-D-quinovopyranosyl) glycerol and 1-*O*-palmitoyl-3-*O*-(6′-sulfo-α-D-quinovopyranosyl) glycerol [34], etc. [35], have been isolated from various marine macroalgae. However, glycolipids from macroalgae are not widely used in the industries such as food and cosmetics. It is important to know that some synthetic glycolipids, such as sugar esters, have been used in food [36]. As a class of natural glycolipids, glycolipids derived from macroalgae have incomparable advantages over chemically synthesized glycolipids, such as diverse activities due to more complex structures, superior safety, and easier acceptance by consumers. In order to promote the application of glycolipids from macroalgae, it is necessary to determine the glycolipids content of macroalgae and establish its extraction and isolation process.

In our work, glycolipids of *Bangia fusco-purpurea*, *Gelidium amansii*, *Gloiopeltis furcata*, *Gracilariopsis lemaneiformis*, *Gracilaria* sp. and *Pyropia yezoensis* were first extracted using five different solvents, and it was determined that the yields of six macroalgae extracts were highest using 95% methanol as extraction solvent (Figure 1). Due to the different types of glycolipids in different seaweeds, the extraction solvents of glycolipids were also different. For example, glycolipids of *Gracilaria corticata* were extracted by EtOAc/methanol (1:1, *v*:*v*) [11]. For glycolipids of *Sargassum fulvellum* [37], *Caulerpa racemosa*, *Osmundaria obtusiloba* and *Dictyota menstrualis* [8], CHCl_3_-MeOH (2:1, *v*:*v*) or CHCl_3_-MeOH (1:2, *v*:*v*) was used as the extraction solvent. The extraction solvent of glycolipids *Ishige sinicola* was methanol. Detection of TLC showed that these extracts contained glycolipids, and *Bangia fusco-purpurea*, *Gracilaria* sp. and *Pyropia yezoensis* extracts were likely to contain higher levels of glycolipids (Figure 1). Subsequently, single-factor experiments were used to determine the extraction factors with higher yield of extracts and glycolipids concentration of three macroalgae mentioned above. Results showed that the effects of volume score of methanol, solid–liquid ratio, extraction temperature, extraction time and ultrasonic power on the yield of extracts and glycolipids concentration were broadly similar, that is, with the increases of factor levels, they increased or increased first and then tended to be stable (Figure 2). Among them, the effect of the volume score of methanol on the yield of *Pyropia yezoensis* extracts was reversed, and the yield was always decreasing in the range of 45~95% volume score of methanol; especially when volume score of methanol was 65% and above, this decreasing trend was particularly obvious (Figure 2). This phenomenon should be related to the polarity of glycolipids in *Pyropia yezoensis* extracts, suggesting that the polarity of glycolipid(s) was likely to be low, and further research will be needed to prove this hypothesis.

*Bangia fusco-purpurea*, *Gracilaria* sp. and *Pyropia yezoensis* extracts showed scavenging activities against DPPH free radicals (Figure 3), and their scavenging abilities were better than that of fucoidan from *Sargassum fusiforme* [36], especially at relatively low concentrations. Namely, at 0.8 g/L, the scavenging activity of *Bangia fusco-purpurea* extracts against DPPH free radicals reached 21.17%, but that of fucoidan from Sargassum fusiforme reached about 20% when its concentration exceeded 1.5 g/L; at the extracts concentration of 3.2 g/L, *Gracilaria* sp. and *Pyropia yezoensis* were close to or above 40% (Figure 3), but that of fucoidan from *Sargassum fusiforme* was about 30% [38]. Three macroalgae extracts were currently used in the antioxidant in vitro experiments, and when these extracts are isolated, the scavenging abilities of some components isolated from macroalgae extracts will increase. This hypothesis was confirmed in Figure 10, where the scavenging abilities of H_12_ and H_33_ against DPPH free radicals were increased by 1.68–3.35 times compared to the *Bangia fusco-purpurea* extracts.

Among the extracts of three macroalgae, *Bangia fusco-purpurea* extracts exhibited good moisture-absorption and moisture-retaining activities (Figure 4). In the current experiments, the moisture-absorption activity of *Bangia fusco-purpurea* extracts was significantly better than that of many seaweed extracts, such as polysaccharides of *Enteromorpha linza* [39], fucoidan sulfate and its derivatives [39] and polysaccharides extracts of five macroalgae (*Saccharina japonica*, *Porphyra haitanensis*, B*ryopsis plumose*, *Codium fragile* and *Enteromorpha linza*) [40]. Also, the moisture-retaining activity of *Bangia fusco-purpurea* extracts was better than that of the polysaccharides extracts of *Saccharina japonica*, *Porphyra haitanensis*, *Bryopsis plumose*, *Codium fragile* and *Enteromorpha linza* [40]. In fact, sugar esters are food additives recommended by the Food and Agriculture Organization of the United Nations and are widely used in food [41]. In Japan, sugar esters are widely used in the preservative application of canned foods [36]. When fresh fruits and vegetables are soaked in sugar esters and dried, a thin film will appear on their surface to retain moisture and improve freshness. The moisturizing effect of *Bangia fusco-purpurea* extracts on the apple pieces has also been discovered in this work (Figure 5). Compared with a previous report [36], the difference was that no film was found on the surface of the apple pieces, which may be have been due to the low concentration of glycolipids in the extracts. In the moisturizing experiment on fresh apple pieces, *Bangia fusco-purpurea* extracts exerted moisturizing effects while exerting antioxidant activity, so the browning of apple pieces was significantly lower than that of the control (Figure 5). Further, the antimicrobial activity of *Bangia fusco-purpurea* extracts was analyzed, and it was found that the extracts had no inhibitory activity against *Escherichia coli*, *Shewanella putrefaciens* and *Bacillus cereus* at the relatively high concentrations (6.4 mg/mL and 25 mg/mL) (Figure 6). This indicates that they need to be used in conjunction with some preservatives when *Bangia fusco-purpurea* extracts are used directly as a moisture-absorption and moisture-retaining agent.

Through comprehensive analysis of the yield and glycolipids concentration of extracts, the antioxidant in vitro, the moisture-absorption and moisture-retaining activity, *Bangia fusco-purpurea* extracts were selected as the target for subsequent experiments (Table 1). For *Bangia fusco-purpurea*, the yield and glycolipids concentration of extracts showed similar changes with the increase of factor levels (Figure 2 and Table 5), so the yield of extracts was determined as the response value of the response surface experiments (Table 2, Table 3 and Table 6). The optimized extraction process of *Bangia fusco-purpurea* was obtained by the response surface experiments (Figure 7), i.e., the solid–liquid ratio, extraction temperature, extraction time and ultrasonic power were, respectively, 1:27 g/mL, 48 °C, 98 min and 450 W. The yield (28.09%) of extracts was 1.1-times higher than the maximum value (25.57%) of the single-factor experiments, but glycolipids concentration increased from 28.96% of the maximum value of the single-factor experiments to 31.57%. In our previous study [12], the glycolipids concentrations of *Codium fragile*, *Neoporphyra haitanensis*, *Sargassum fusiforme*, *Saccharina japonica*, *Silvetia siliquosa* and *Undaria pinnatifida* extracts were also determined, and concentrations were significantly lower than that of *Bangia fusco-purpurea* extracts. The result indicates that *Bangia fusco-purpurea* extracts were an ideal source of glycolipids. In addition, *Gracilaria* sp. extracts also showed some moisture-absorption and moisture-retention activity (Figure 4), significant scavenging activities against DPPH free radicals (Figure 3) and a higher yield of extracts. Therefore, the extraction process and isolation of the antioxidant active components from *Gracilaria* sp. extracts can be studied in the follow-up work.

Although glycolipids were widely present in living organisms, their contents were very low, and it was difficult to isolate and purify of glycolipids. In this paper, three samples were prepared by a series of isolation and purification steps from *Bangia fusco-purpurea*, namely liquid–liquid extraction (Table 4 and Figure 8), silica gel column chromatography (Figure 9) and preparation thin-layer chromatography (Supplementary Material, Figure S2). In the preliminary isolation of glycolipids, ion-exchange chromatography and silica gel column chromatography are commonly used methods. Glycolipids were preliminarily isolated from *Ishige sinicola* by ion exchange (Diaion HP-20) chromatography [40]. The difference between the same type of glycolipids is only reflected in the composition of the acyl fatty acids, but the molecular chargeability is very close, so some studies prefer to use silica gel column chromatography to isolate glycolipids, such as *Chondria armata* [5], *Gracilaria corticata* [11], *Laminaria japonica* [42], *Ulva lactuca* [43] and *Sargassum fulvellum* [36], etc. Since the adsorption capacity of silica gel to glycolipids is related to the properties of the glycolipids themselves, the eluents used for silica column chromatography vary widely between different types of glycolipids from macroalgae. Akbari et al. reported that the eluent for silica gel column chromatography isolation of glycolipids of *Gracilaria corticata* was a gradient solvent system from 100% n-hexane to pure EtOAc [11]. For the silica gel column chromatography isolation of glycolipids of *Ulva lactuca*, the composition of the eluents was more complex. In order, the compositions were acetone, acetone/benzene/acetic acid/water (200:30:3:10, *v*:*v*:*v*) and a gradient of chloroform and methanol [43]. In the study of glycolipids of *Dictyota menstrualis*, *Osmundaria obtusiloba* and *Caulerpa racemosa*, the eluents for silica gel column chromatography were chloroform, acetone and then methanol [8,43]. Petroleum ether, petroleum ether: ethyl acetate (1:4), dichloromethane, dichloromethane: methanol (2:3) and methanol were sequentially used as the solvents for silica gel column chromatography in the isolation of glycolipids of *Bangia fusco-purpurea*. These obtained fractions were often analyzed by TLC during the isolation by the silica gel column chromatography [1,43]. In our work, the glycolipids of six macroalgae extracts’ (Figure 1) portions (Figure 8) extracted from *Bangia fusco-purpurea* extracts and fractions isolated by silica gel column chromatography were also detected by TLC.

Although silica gel column chromatography can process a large number of samples, it has some disadvantages of serious irreversible adsorption, low isolation efficiency and large solvent consumption, so it is necessary to find more advantageous resins to isolate glycolipids. After the initial isolation of the glycolipids from macroalgae, these glycolipids were further purified or prepared by Sephadex LH-20 gel column chromatography [5], or silica gel column chromatography [37,44], and/or preparative thin layer chromatography [11,41,45]. Finally, two glycolipids were isolated and purified from *Bangia fusco-purpurea* (Figure 9). This was the first isolation of glycolipids from *Bangia fusco-purpurea* [12]. Up to now, there has not been many studies on the extraction, isolation and purification of glycolipids from macroalgae [2,8,12]. In view of the great application potential of glycolipids with multi activity in the medicine, food and other fields, more glycolipids from marine macroalgae need to be isolated and purified.

Two glycolipids H_12_ and H_33_ prepared by our work showed good antioxidant activity in vitro and/or moisturizing activity (Figure 10). The researches pointed out that the moisture-absorption and moisture-retention mechanism of seaweed polysaccharides is as follows: (1) Seaweed polysaccharides contain a large number of hydrophilic groups, such as hydroxyl and carboxyl, which can be combined with water molecules in the form of hydrogen bonds [46]. (2) Algal polysaccharide molecular chain and water molecules can be crosslinked and wound in space to form a network structure [47]. According to the composition of glycolipids, they should also have the moisture-absorption and moisture-retention activities. The results of this paper confirm this view. *Bangia fusco-purpurea* extracts and the purified sample H_33_ exhibited good moisture-absorption and moisture-retention activities (Figure 4, Figure 5 and Figure 10). The reason why they have moisture-absorption and moisture-retention activity can be concluded according to the structure of H_33_ (Figure 9). The long chain of hydrophilic end of glycolipid can combine with water molecules in the air to maintain the water content of the sample and play the moisture-absorption and moisture-retention role.

Unfortunately, due to insufficient preparation quantity, the antimicrobial activity of H_12_ and H_33_ could not be determined in this paper. At present, it could only be determined that *Bangia fusco-purpurea* extracts did not exhibit antimicrobial activity at relatively high concentrations. Several reports have pointed out that glycolipids containing medium-chain fatty acids (C10, C12) have good antibacterial effects [48], and glycolipids with long-chain fatty acids (C16, C18) have the worst antibacterial effects [29]. According to these studies, the fat chains of the two glycolipids H_12_ and H_33_ are likely to be long and do not have antibacterial activity. Two glycolipids, H_12_ and H_33_, will be necessary to identify the structure and determine whether have antibacterial activity in the follow-up work.

## 4. Materials and Methods

### 4.1. Marine Macroalgae

*Bangia fusco-purpurea* (Rhodophyta), *Gelidium amansii* (Rhodophyta), *Gloiopeltis furcata* (Rhodophyta), *Gracilariopsis lemaneiformis* (Rhodophyta), *Gracilaria* sp. (Rhodophyta) and *Pyropia yezoensis* (Rhodophyta) semi-dried products were purchased from the wholesalers. Except for *Pyropia yezoensis*, which was purchased from Lianyungang, other macroalgae were purchased from a wholesaler Fujian Wangduofu Biotechnology Co., Ltd. in Xiapu, Fujian, China. In addition, 500 g of marine macroalgae were quickly rinsed with distilled water, blotted on tissue papers and dried in a blast drying oven at 40 °C for 12 h. Dried macroalgae materials were cut into small pieces (ca. 2.0 cm of length) or crumbled. And then these pieces were ground to make powder using a blender for 1 min. The powder was conserved at −20 °C until the extraction.

### 4.2. Chemical Reagents

Monogalactosyldiacylglycerols (MGDG), digalactosyldiacylglycerols (DGDG) and sulfoquinovosyl diacylglycerol (SQDG) standards were obtained from Avant Polar Lipids (USA). The other solvents or compounds were analytically pure and from Sinopharm Chemical Reagent Co., Ltd., in Shanghai, China.

### 4.3. Extraction

#### 4.3.1. Determination of Different Extraction Solvents and Extraction Time

Firstly, the effects of five extraction solvents (95% ethyl acetate, dichloromethane: methanol (3:2), 95% ethanol, 95% methanol and acetone) on the yields of six macroalgae extracts were analyzed to obtain suitable extraction solvent. Then, 10 g powder of macroalgae was poured into 300 mL of each extraction solvent and placed on an ultrasonic cleaner for ultrasonic extraction twice under the conditions of a solid-to-liquid ratio of 1:30 g/mL, temperature of 45 °C, extraction time of 180 min and extraction power of 500 W. After the extraction was completed, the leach liquor was filtered and concentrated under reduced pressure to obtain macroalgae extracts. The yield of extracts was calculated according to the formula:

Yield (%) = 100% × extracts quantity/macroalgae powder quantity(2)

Subsequently, in order to obtain the appropriate extraction time, the effects of extraction times (1, 2, 3 and 4) on the yields of six macroalgae extracts were also analyzed. The extraction process was the same as the above-mentioned process.

#### 4.3.2. Single-Factor Experiments

First, 10 g powder of macroalgae was poured into 300 mL or a set volume of methanol solution (volume score) and placed on an ultrasonic cleaner for ultrasonic extraction for a certain time under different ultrasonic power and temperature. After filtration, we repeated the above operation twice for the filter residue. The filter solution was combined and evaporated to dryness under reduced pressure. The extraction process of the extracts containing glycolipids was optimized. In this process, the solid-to-liquid ratio, extraction temperature, time, ultrasonic power and volume score of methanol were set according to Table 1. When conducting the solid–liquid ratio experiments, the extraction temperature, time, ultrasonic power (XO25-12DTS (25 KHZ) Ultrasonic Cleaner, Nanjing AtPio Instrument Manufacturing Co., Ltd., Nanjing, China) and volume score of methanol were set to 45 °C, 120 min, 500 W and 85%. In the extraction temperature experiments, the solid–liquid ratio with higher yield of extracts and the glycolipids concentration in the solid-to-liquid ratio experiments was selected as the value of the solid–liquid ratio, while the extraction time, ultrasonic power and volume score of methanol were set to 120 min, 500 W and 85%. For the extraction time experiments, the ultrasonic power and volume score of methanol were set to 500 W and 85%, but the solid–liquid ratio and extraction temperature were set according the results of the solid-to-liquid ratio experiments and the extraction temperature experiments, namely the solid–liquid ratio and extraction temperature with higher yield and glycolipids contents in the solid–liquid ratio experiments and the extraction temperature experiments were selected. Other factor experiments were performed according to same methods. In this way, the extracts of marine macroalgae were obtained. After weighing the mass of the distillation flask and residue, 10 mL of 10% ethanol solution was added to dissolve the residue and obtain the extracts solution. The extracts solution was subject to acid hydrolysis treatment according to the method in Section 4.4.2. And then, the absorbance of the hydrolysate at 480 nm was measured, and w obtained the concentration of glycolipids according to the standard curve mentioned in Section 4.4.2. Furthermore, the quantity of glycolipids could be obtained.

#### 4.3.3. Response Surface Methodology

On the basis of the results of the single-factor experiments, with factors A (solid-to-liquid ratio), B (temperature), C (time) and D (ultrasonic power) as the independent variables and the yield as the response value, the Box-Behnken model was used to design the four-factor and three-level of response surface analysis experiment with a total of 29 test points. The Design-Expert 11 software was used to perform regression analysis on the experimental data to determine the optimal extraction process. The three levels for each factor are shown in Table 6. The Design-Expert 11 software was used for response surface method experiment design and analysis. In addition, 10 g powder of macroalgae was treated using methods in Section 4.3.2.

### 4.4. Qualitative and Quantitative Detection of Glycolipids

#### 4.4.1. Qualitative Detection

Glycolipids of macroalgae extracts were determined by silica gel thin-layer chromatography. First, 0.1 g of macroalgae extracts were dissolved in 20 mL of methanol and diluted 10 times to obtain the extracts’ solution. These extracts’ solution and three standards (MGDG, DGDG, SQDG) were, respectively, spotted on the chromatographic plate, developed with chloroform: methanol: water (65:15:3, *v*:*v*:*v*), sprayed phenol-sulfuric acid for color development and dried at 110 °C for 20 min. The positive reaction of glycolipids was to present brownish spots.

#### 4.4.2. Quantitative Detection

First, 1 mL of 5% phenol and 5 mL of concentrated sulfuric acid were added to 2 mL glycolipids solution successively, and then a water bath was taken at 80 °C for 30 min. After cooling to room temperature, the absorbance of the hydrolysate was measured at 480 nm (or 490 nm). According to the standard curve, the concentration of glycolipids of extracts was calculated, so the quantity of glycolipids was acquired. On this basis, glycolipids concentration was determined according to the following formula:

Glycolipids concentration (%) = 100% × glycolipids quantity/extracts quantity(3)

D-arabinose was prepared with 10% ethanol into a solution with a mass concentration of 10, 20, 30, 40, 50, 60, 70, 80 and 90 µg/mL. 1 mL of phenol was added to 2 mL of each concentration of solution, and then 5 mL of concentrated sulfuric acid was quickly added, shook to mix evenly and placed in an 80 °C constant temperature water bath for 30 min. After cooling to room temperature, the absorbance of 2 mL of the above hydrolysate at 480 nm was measured. 10% ethanol was as the blank control group. According to the above data, the regression equation between the mass concentration (*X*) of arabinose and absorbance (*Y*) of D-arabinose solution was established, namely *Y* = 0.0107*X* + 0.1282 (*R*^2^ = 0.995).

#### 4.4.3. Silica Gel Thin-Layer Chromatography Detection

The standards (MGDG, DGDG, SQDG), macroalgae extracts, portions, eluents and prepared samples were, respectively, spotted on the chromatographic plate, developed with chloroform: methanol: water (65:15:3, *v*:*v*:*v*), sprayed phenol-sulfuric acid for color development and dried at 110 °C for 20 min, or developed with iodine vapor.

### 4.5. Isolation and Purification

Two portions of glycolipids extracts (4.0 g) were taken and dissolved in 50 mL of distilled water and buffer solution with pH 2. The preparation method of buffer solution was as follows: 3.7275 g KCl and 25.00 g NaCl were dissolved in 200 mL of distilled water and poured into a 1000 mL of volumetric flask. Then, 6.5 mL of 2 mol/L hydrochloric acid solution was added to adjust the pH to 2 and fix the volume to 1000 mL, respectively. And then, th mixed solutions mentioned above were, respectively, portioned using different solvents according to the following steps.

The mixed solution of distilled water (or buffer solution) was poured into the separating funnel. Then, 25 mL of hexane was added and vibrated fully, to stand for 2~4 h, and the upper phase was collected. This process was repeated 3 times. All the upper phases were combined and evaporated under reduced pressure to obtain the hexane portion (HP), and the remaining phase was extracted with dichloromethane 3 times. After the extraction with dichloromethane was completed, ethyl acetate was used for extraction according to the above method. Finally, n-butanol was added for extraction. According to the liquid–liquid extraction method, the hexane portion (HP), dichloromethane portion (DP), ethyl acetate portion (EP) and n-butanol portion (BP) were obtained in turn. Three parallel samples were set for each of the above experimental treatments. After weighing the quantity, these portions were dissolved in 95% methanol and prepared to the concentration of 5 mg/mL. TLC was used to test these portions for glycolipids and compared to the behavior of standard glycolipids (MGDG, DGDG and SQDG) in silica gel plate. And then target portion containing glycolipids was isolated by silica gel column chromatography (100–200 mesh, 4.0 × 40 cm). Petroleum ether, petroleum ether: ethyl acetate (1:4), dichloromethane, dichloromethane: methanol (2:3) and methanol were used as eluents in turn with a flow rate of 1.0 mL/min. After eluting the column volume twice, the next eluent was switched. Subsequently, all eluents were evaporated at 40 °C under reduced pressure, and then the resulting fractions were combined according to the behavior in silica gel TLC. These target fractions were further purified by preparation TLC, with chloroform: methanol: water (65:15:2, *v*:*v*:*v*) used as the developing agent. The target fraction was streaked on 2 silica gel plates (G1 and G2) and unfolded. Among them, one G plate (G1) was sprayed with 20% concentrated sulfuric acid–ethanol solution, and the other (G2) was scraped according to the development of G1 to obtain purified sample. Finally, the prepared sample was compared with Rf of TLC and the residence time of HPLC of the standard.

### 4.6. Antioxidant, Moisture-Absorption and Moisture-Retention, and Antibacterial Activity Assay

#### 4.6.1. The Scavenging Activity on DPPH Free Radicals

The scavenging activity of macroalgae extracts was analyzed on DPPH (2, 2-Diphenyl-1-picrylhydrazyl) using the method reported by Sun et al. [49] with some modifications. The concentrations of macroalgae extracts were set to 400, 800, 1600 and 3200 µg/mL, and Vitamin C (Vc), used as the positive control, was set as 100, 200, 400, 800, 1600 and 3200 μg/mL. The absorbance (*A_e_*) of the above reaction solution was determined at a wavelength of 517 nm. The concentration of the same amount of ultrapure water (substitute macroalgae extracts) and 2 mL of the 0.1 mmol/L DPPH ethanol solution were mixed and reacted in dark for 30 min for the blank groups, and the absorbance was recorded as *A_1_*. For the control groups, the DPPH solution with an equal amount of ethanol was used; then, the above operation was performed, the absorbance was record *A*_2_. Three parallel samples were set in these experiments. The scavenging activity of tested samples on DPPH free radicals can be calculated according to Equation (4):

(4) $$\mathrm{The}\;\mathrm{scavenging}\;\mathrm{activity}\;\mathrm{on}\;\mathrm{DPPH}\;\mathrm{free}\;\mathrm{radicals}\;(\%)=1-\frac{A_{e}-A_{2}}{A_{1}}\times 100\%$$

where *A*_2_ is the absorbance of the control, *A_e_* is the absorbance of tested sample and *A*_1_ is the absorbance of the blank only.

The scavenging activity on DPPH free radicals of the purified sample isolated from macroalgae extracts was also measured according to the above method, and the concentration was set to 100, 200, 400, 800, 1600 and 3200 μg/mL.

#### 4.6.2. The Total Antioxidant Capacity

The purified sample was prepared as a sample solution with a concentration of 0.25 mg/mL for backup. The ABTS working solution, reagent IV application solution and Trolox solution were prepared with different concentrations according to the ABTS kit method (Nanjing Jiancheng Biotechnology Research Institute). Then, 170 μL of the ABTS working solution was added to each well plate of the microplate reader, and 10 μL of methanol was added to the blank well plate as a control. In addition, 10 μL of the sample solution was added to the measurement well, and standard solutions with different concentrations were added to the standard well. Then, we immediately covered the well plate cover and react indoors for 6 min. Then, the absorbance at 405 nm was measured by the microplate reader. The total antioxidant capacity of the sample was calculated based on the Trolox standard curve (the regression equation was *y* = −1.1897*x* + 1.7403 (*R*^2^ = 0.9911; *x* was the absorbance at 405 nm, *y* was Trolox concentration (mM)) and the total antioxidant capacity was expressed using Trolox Equivalent Antioxidant Capacity (TEAC, mmol/mg).

#### 4.6.3. Moisture-Absorption Measurement

We referred to the method in reference [50], wherein 0.5 g of sodium alginate, sorbierite, glycerin and macroalgae extracts, which were previously dried to a constant weight, were accurately weighed, respectively, and put in Petri dishes with a diameter of 5 cm. Then, these Petri dishes were placed in the dryer. There was saturated sodium carbonate solution in the dryer (the relative humidity (RH) was 43%). The placement time was set to 2 h, 4 h‚ 8 h, 12 h, 24 h, 36 h, 48 h, 60 h and 72 h, and the moisture-absorption rate was calculated according to the mass difference of samples before and after placement. According to the same method mentioned above, the moisture absorption test of trehalose, sodium alginate, sorbitol, glycerol and macroalgae extracts at a RH of 81% (saturated ammonium sulfate solution in the dryer) was carried out.

Moisture-absorption rate = [(m_n_ − m_0_)] × 100% (5)

where m_0_ and m_n_ were the mass of test sample before and after placement, respectively.

#### 4.6.4. Moisture-Retention Measurement

The method of the literature [40] was taken, and it was improved. First, the tested sample (trehalose, sodium alginate, sorbitol, glycerol and macroalgae extract) was prepared with a concentration of 0.1 mg/mL. Then, 0.6 g of tested sample solutions were accurately weighed and put into weighing bottles, respectively. These weighing bottles were placed in a dryer with dry silica gel for 2 h, 4 h, 8 h, 20 h, 30 h, 42 h, 54 h, 66 h and 72 h, and the moisture-retention rate was calculated according to the mass difference of samples before and after placement. The moisture-retention of the purified sample isolated from macroalgae extracts was also measured according to the above method.

Meanwhile, the moisturizing effects of macroalgae extracts on apple pieces were designed. Each apple was cut into 12 pieces, the thickness of each apple piece was controlled to 2.5 mm ± 0.4 mm, and the quantity was controlled to 12.0 g ± 0.5 g. In order to exclude individual differences between the apple pieces, three apple pieces in same experimental group were taken from three apples, respectively. Fresh apple pieces were soaked in extracts solution (or distilled water) for 1 min. Then, they were removed, and the quantity of the apple pieces (H_0_) was measured. Subsequently, these apple pieces were placed for 4 h, 8 h, 20 h, 36 h, 48 h, 60 h and 72 h at room temperature. At each set time point, these apple pieces were removed, and the quantity was measured (H_n_). (The Petri dish containing the apple pieces was removed, and the quantity was measured. The room temperature was kept at 25 °C.) The moisture-retention rate was calculated using Equation (6).

Moisture-retention rate = (H_n_/H_0_) × 100% (6)

where H_0_ and H_n_ were the mass of test sample before and after placement, respectively.

#### 4.6.5. Antibacterial Activity Measurement

*Escherichia coli*, *Shewanella putrefaciens* and *Bacillus cereus* are food-borne pathogens. First, 0.05 g of macroalgae extracts was weighed, and it was prepared with methanol at concentrations of 25 mg/mL and 6.4 mg/mL (in a compound Bromo Geraminum Disinfectant (Shandong Retouch Washing and Disinfection Technology Co., Ltd., Dezhou, Shandong). The concentration of the inhibitory component was set to 25 g/L. In a compound antibacterial agent [50], the concentration of the inhibitory component was set at 6.4 g/L), respectively. They were then individually coated onto the LB medium [48]. The LB medium was packed in multiple 90 mm diameter Petri dishes. Multiple 5 mm holes in LB medium coated with test bacteria were punched using the hole punch, 100 μL of tested extracts were added, and 50 μL of ultrapure water was added as a control. Subsequently, these Petri dishes were incubated in a biochemical incubator at 37 °C for 16 h. The average value of the inhibition zone was used as the final antibacterial activity index. The experimental group with the addition of macroalgae extracts was set up with 8 parallel samples, and the control group was set up with 5 parallel samples.

### 4.7. Data Processing and Statistical Analysis

Unless otherwise specified, each experiment was set up with 3 parallel samples. The data obtained are expressed as the mean ± SD. All the data of the growth assays in this study were analyzed by ANOVA using SPSS 27.0.1 (in single-factor experiments, ANOVA was also performed but not listed).

Design-Expert 11 software was used to analyze the results of Response Surface Methodology.

## 5. Conclusions

Due to their important role in many biological and pathological processes, glycolipids have been of interest for a long time [1,4,5,7], despite their low content in marine macroalgae [12,51]. In the present study, the extracts of *Bangia fusco-purpurea*, *Gelidium amansii*, *Gloiopeltis furcata*, *Gracilariopsis lemaneiformis*, *Gracilaria* sp. and *Pyropia yezoensis* were obtained to the detection of glycolipids. A series of single-factor and response surface experiments were used to establish the extraction process of glycolipids of Bangia fusco-purpurea. When the solid–liquid ratio, extraction temperature, extraction time and ultrasonic power were 1:27 g/mL, 48 °C, 98 min and 450 W, respectively, the average yield of the extracts was the highest, which was 28.09%. It was found that *Bangia fusco-purpurea* extracts had a certain scavenging effect on DPPH free radicals, as well as good moisture–absorption and moisture-retaining activities. Two purified samples were finally prepared from *Bangia fusco-purpurea* by liquid–liquid extraction, silica gel column chromatography and thin-layer chromatography. This is the first time that the two glycolipids have been isolated from *Bangia fusco-purpurea*. These two glycolipids showed good antioxidant activity in vitro and (or) moisturizing activity, and the other activity and activity mechanisms of them will be studied in the follow-up study. And in order to promote the application of glycolipids from macroalgae, the extraction and isolation of glycolipids will be carried out around more species of macroalgae. The results of this study provide a valuable basis for the development of glycolipids functional products in food, cosmetics, medicine and other industries.

## Figures and Tables

**Table 1:** Results of glycolipids concentration, yield, and moisture-absorption and moisture-retaining rates of three macroalgae extracts.

		*Bangia fusco-purpurea*	*Gracilaria* sp.	*Pyropia yezoensis*
Maximum Glycolipids concentration (%) (the yield (%) at the certain conditions)		28.959 ± 1.542 (25.573 ± 1.031 at volume score of methanol, solid–liquid ratio, extraction temperature, extraction time and ultrasonic power were 85%, 1:25 mg/L, 45 °C, 120 min and 500 W.)	27.439 ± 1.071 (33.303 ± 1.893 at volume score of methanol, solid–liquid ratio, extraction temperature, extraction time and ultrasonic power were 85%, 1:30 mg/L, 55 °C, 120 min and 500 W.)	26.549 ± 1.011 (26.529 ± 1.153 at volume score of methanol, solid-–iquid ratio, extraction temperature, extraction time and ultrasonic power were 55%, 1:30 mg/L, 45 °C, 120 min and 500 W.)
Moisture absorption rate (%) after 48 h	RH81%	51.968 ± 2.441~55.425 ± 3.122	30.620 ± 2.443~32.701 ± 2.261	26.997 ± 1.512~29.094 ± 2.154
	RH43%	39.279 ± 1.563~46.328 ± 1.794	31.489 ± 2.781~38.734 ± 3.362	17.340 ± 1.533~19.436 ± 2.250
Moisture retaining rates (%) after 48 h		74.046 ± 1.350	73.192 ± 1.383	72.954 ± 1.152

Note: The data in the figure are expressed as the mean ± SD.

**Table 2:** Box-Behnken experimental design and results.

No.	Solid–Liquid Ratio (g/mL): A	Extraction Temperature (°C): B	Extraction Time (h): C	Ultrasonic Power (W): D	Yield (%)
1	30	45	60	450	26.32
2	30	35	90	450	26.20
3	20	45	90	500	27.12
4	25	35	90	500	26.31
5	25	55	60	450	26.85
6	25	45	120	500	27.06
7	25	45	90	450	28.05
8	25	55	90	500	28.05
9	20	55	90	450	26.45
10	25	45	60	500	27.58
11	30	55	90	450	27.36
12	20	35	90	450	25.75
13	25	45	90	450	27.75
14	25	35	90	400	26.75
15	25	35	120	450	26.28
16	20	45	120	450	26.17
17	25	35	60	450	25.78
18	25	55	120	450	27.35
19	25	45	120	400	27.49
20	25	45	60	400	26.48
21	25	55	90	400	27.13
22	30	45	120	450	27.38
23	20	45	60	450	26.28
24	25	45	90	450	27.82
25	30	45	90	500	27.36
26	30	45	90	400	27.46
27	25	45	90	450	27.79
28	25	45	90	450	27.84
29	20	45	90	400	26.26

**Table 3:** Significance testing and analysis of variance for regression equation models of the yield of extracts.

Source	Sum of Squares	df	Mean Squares	*F*-Value	*p*-Value	Significant
Model	13.54	14	0.9675	110.35	<0.0001	Significant
A	1.36	1	1.36	155.68	<0.0001	**
B	3.11	1	3.11	355.21	<0.0001	**
C	0.4998	1	0.4998	57.01	<0.0001	**
D	0.3082	1	0.3082	35.15	<0.0001	**
AB	0.0515	1	0.0515	5.88	0.0295	*
AC	0.3405	1	0.3405	38.84	<0.0001	**
AD	0.2304	1	0.2304	26.28	0.0002	*
BC	0.0000	1	0.0000	0.0010	0.9749	
BD	0.4713	1	0.4713	53.76	<0.0001	**
CD	0.5852	1	0.5852	66.75	<0.0001	**
A^2^	3.28	1	3.28	374.54	<0.0001	**
B^2^	3.12	1	3.12	355.58	<0.0001	**
C^2^	2.32	1	2.32	264.36	<0.0001	**
D^2^	0.0579	1	0.0579	6.61	0.0222	*
Residual error	0.1227	14	0.0088			
Lack of fit	0.0660	10	0.0066	0.4646	0.8516	Not significant
Pure error	0.0568	4	0.0142			
Total dispersion square sum	13.67	28				
*R*^2^ = 0.9910	*R*_Adj_^2^ = 0.9820					

Note: “**” represents a very significant difference, *p* < 0.01; “*” represents a significant difference, *p* < 0.05.

**Table 4:** The portion of *Bangia fusco-purpurea* extracts.

Portion	Extracts Were Dissolved in Distilled Water		Extracts Were Dissolved in Buffer Solution with pH 2	
	Yield (%)	Quatity (g)	Yield (%)	Quatity (g)
Hexane portion (HP)	0.805 ± 0.275	0.0322 ± 0.011	2.407 ± 0.725	0.0963 ± 0.029
Dichloromethane portion (DP)	3.050 ± 0.400	0.122 ± 0.016	3.775 ± 0.950	0.151 ± 0.038
Ethyl acetate portion (EP)	1.770 ± 0.425	0.0708 ± 0.017	1.880 ± 0.525	0.0752 ± 0.021
N-butanol portion (BP)	8.725 ± 1.925	0.349 ± 0.077	7.425 ± 2.125	0.297 ± 0.085

Note: The data in the figure are expressed as the mean ± SD.

**Table 5:** Factors and levels of single-factor experiments.

	Factor	Volume Score of Methanol: (%)	Solid-to-Liquid Ratio (g/mL)	Temperature (°C)	Time (min)	Ultrasonic Power (W)
Level						
1		45	1:10	25	30	250
2		55	1:15	35	60	300
3		65	1:20	45	90	350
4		75	1:25	55	120	400
5		85	1:30	65	150	450
6		95	1:35	75	180	500

**Table 6:** Factors and levels of response surface methodology.

	Factor	Solid-to-Liquid Ratio (g/mL): A	Temperature (°C): B	Time (min): C	Ultrasonic Power (W): D
Level					
1		1:20	35	60	400
2		1:25	45	90	450
3		1:30	55	120	500

## Data Availability

Data are contained within the article and Supplementary Materials.

## Supplementary Materials

The following supporting information can be downloaded at: https://www.mdpi.com/article/10.3390/md22040144/s1, Figure S1: TLC determination of the eluents isolated from dichloromethane phase of Bangia fusco-purpurea extracts through silica gel column chromatography; Figure S2: Preparation of thin layer chromatography of glycolipids from Bangia fusco-purpurea. Figure S3: Nuclear magnetic resonance carbon spectrum of H_12_; Figure S4: Nuclear magnetic resonance hydrogen spectrum of H_12_; Figure S5: Nuclear magnetic resonance carbon spectrum of H_33_; Figure S6: Nuclear magnetic resonance hydrogen spectrum of H_33_.
