# Supplementary material for: Extraction, Isolation and Biological Activity of Two Glycolipids from Bangia fusco-purpurea
Source: Mar Drugs. 2024 Mar 24;22(4):144. doi: 10.3390/md22040144 (PMC11051132; doi:10.3390/md22040144)
Supplement: Supplementary file 1 [file marinedrugs-22-00144-s001.zip › marinedrugs-2889631-supplementary.pdf]

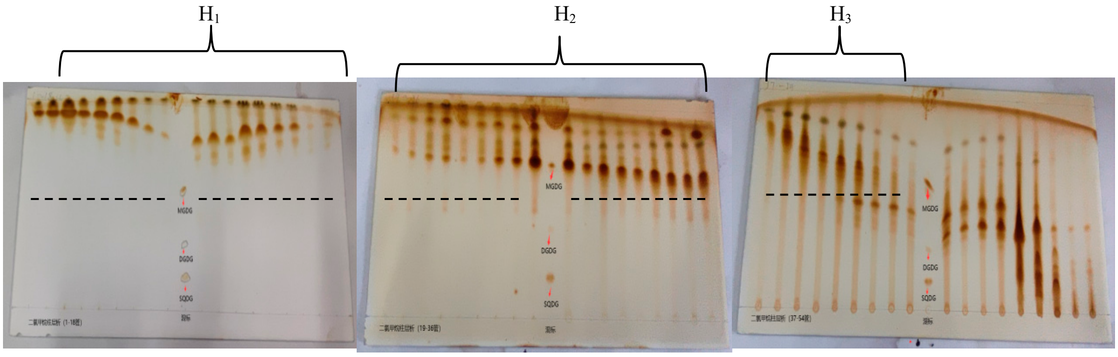

Figure S1: TLC determination of the eluents isolated from dichloromethane phase of *Bangia fusco-purpurea* extracts
through silica gel column chromatography

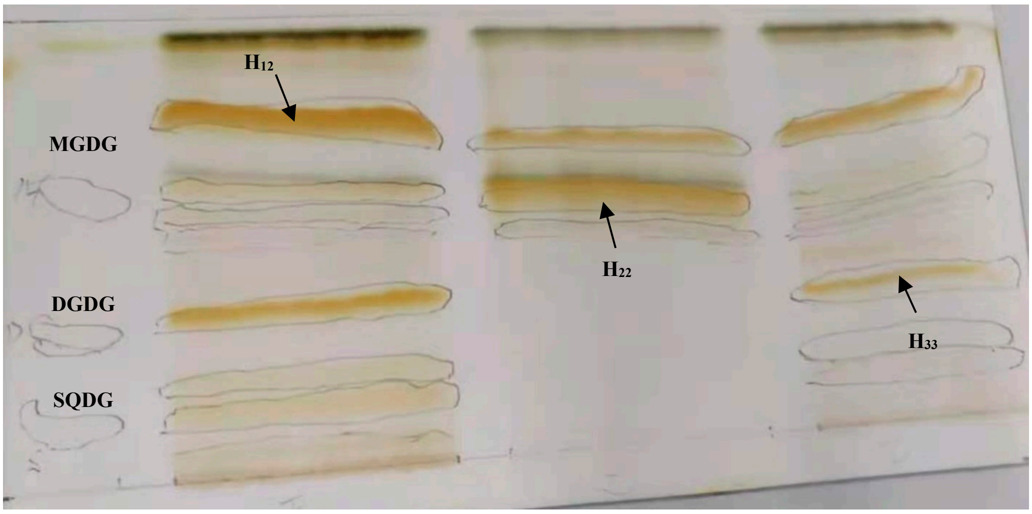

(The strips of H_1, H_2 and H_3 were not in the same positions as that of the three standards MGDG, DGDG and SQDG. MGDG, DGDG and SQDG were as a reference only)

Figure S2: Preparation of thin layer chromatography of glycolipids from *Bangia fusco-purpurea*

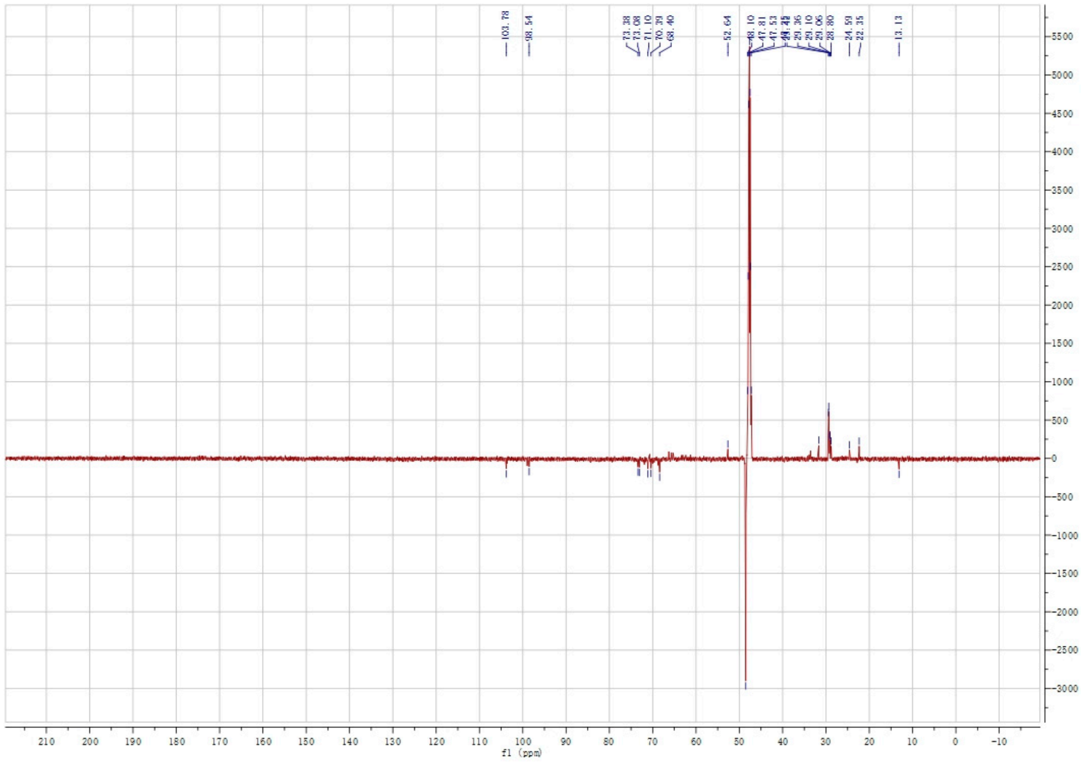

Figure S3: Nuclear magnetic resonance carbon spectrum of H_{12}

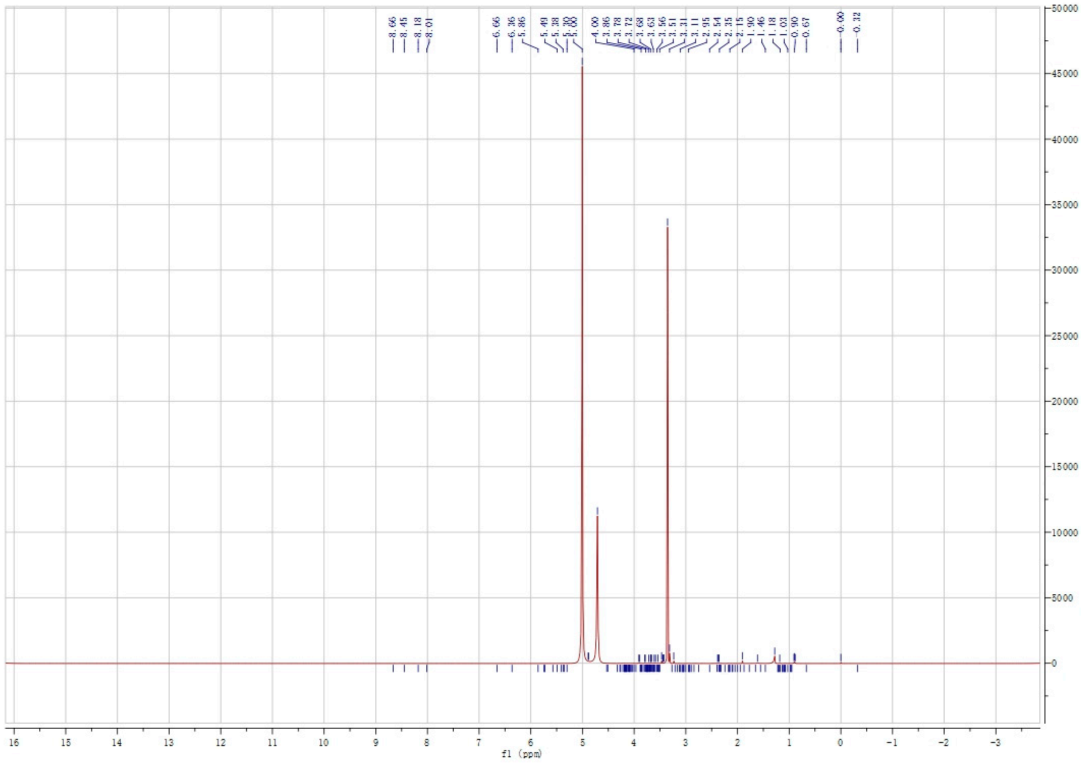

Figure S4: Nuclear magnetic resonance hydrogen spectrum of H_{12}

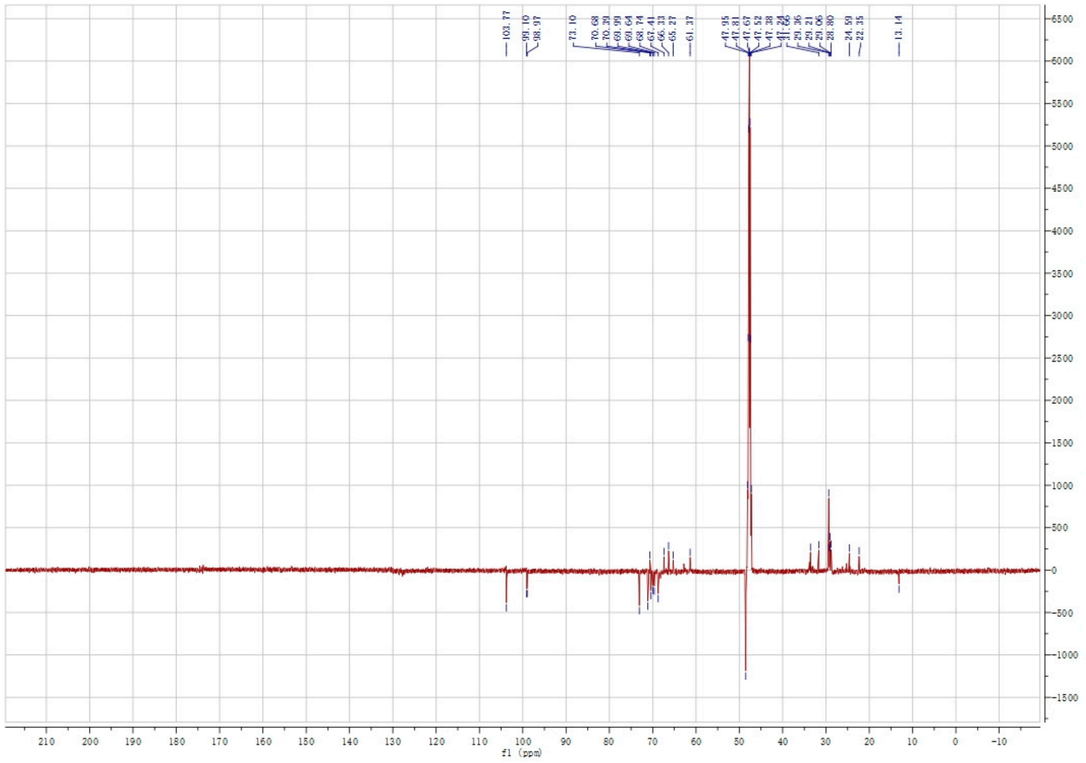

Figure S5: Nuclear magnetic resonance carbon spectrum of H_{33}

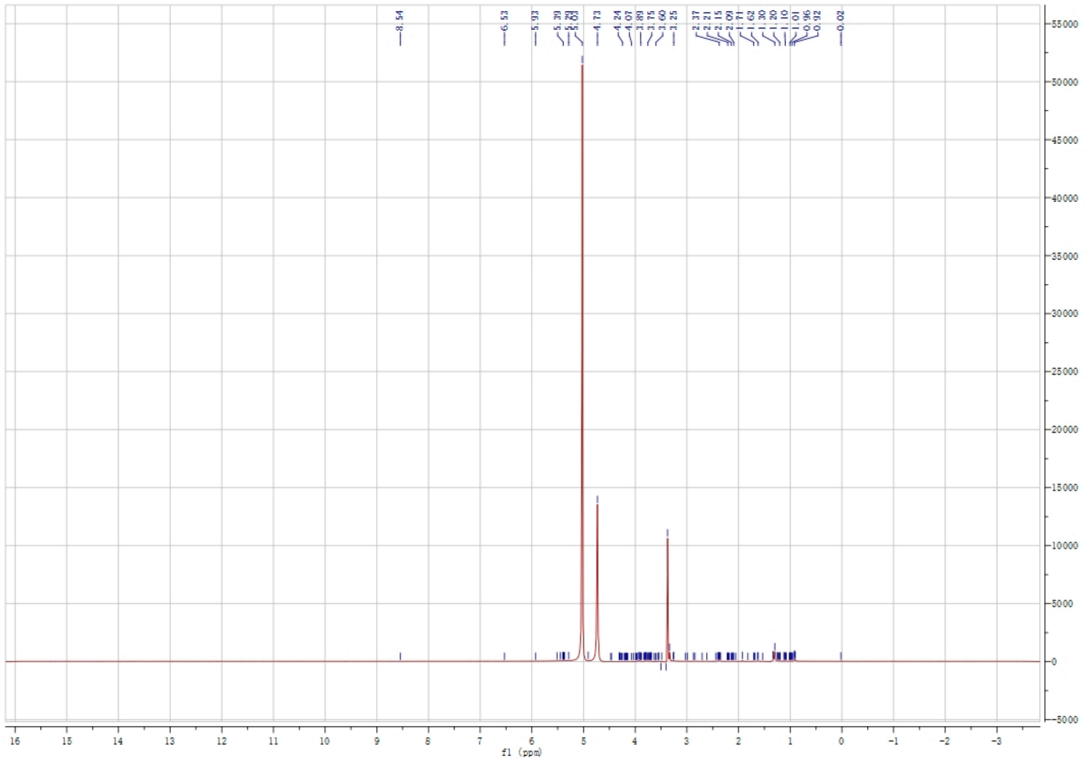

Figure S6: Nuclear magnetic resonance hydrogen spectrum of H_{33}
